# Consumption status of functional drinks based on the theory of planned behavior and the stages of change model in female employees

**DOI:** 10.1038/s41598-024-64888-7

**Published:** 2024-06-20

**Authors:** Azam Toorani, Mitra Moodi, Tayebeh Zeinali, Fatemeh Salmani, Ensiyeh Norozi

**Affiliations:** 1 Student Research Committee, Birjand University of Medical Sciences, Birjand, Iran; 2 Department of Health Education and Promotion, School of Health, Social Determinants of Health Research Center, Birjand University of Medical Sciences, Birjand, Iran; 3 Department of Nutrition and Food Hygiene, School of Health, Infectious Diseases Research Center, Birjand University of Medical Sciences, Birjand, Iran; 4 Department of Epidemiology and Biostatistics, School of Health, Social Determinants of Health Research Center, Birjand University of Medical Sciences, Birjand, Iran; 5 Department of Public Health, School of Health, Social Determinants of Health Research Center, Birjand University of Medical Sciences, Birjand, Iran

**Keywords:** Functional food, Drink, Attitude, Theory of planned behavior, Stage of change model, Nutrition, Public health

## Abstract

This study aimed to determine the consumption status of functional drinks based on the theory of planned behavior and the Stages of Change Model. This study was conducted on 536 female employees of Birjand offices in 2022. The data was collected by using the functional food questionnaire and was analyzed using SPSS with significance level of *P* < 0.05. The largest number of people consume probiotic drinks (buttermilk, kefir, etc.) were in the maintenance stages (31.2%) and who consume milk fortified with vitamin D were in the contemplation stage (37.3%). The mean score of the participants in the subscales of attitude, behavioral control, and subjective norm was 17.69 ± 3.05, 16.83 ± 2.88, 21.73 ± 4.33, respectively. The mean score of the attitude and subjective norm regarding the consumption of all drinks had a significant relation with the stages of change model (*p* < 0.05). The results of this study showed that the most drinks that female employees consumed regularly were probiotic drinks, while they did not intend to use functional juices (pre-contemplation stage). Therefore, it seems that this theory can be used as a framework in designing educational programs in order to increase the consumption of functional foods and improve women's health.

## Introduction

Consumers' awareness of the relationship between food and health has increased their interest in healthy food^1^. Healthy eating prevents most diseases like chronic diseases such as diabetes, cancer and cardiovascular problems^1–3^. Chronic diseases are often caused by an inappropriate diet^4^. The World Health Organization (WHO) estimates that more than two billion people worldwide suffer from micronutrient deficiencies. Recently, foods and drinks have been known as functional foods are used for the prevention and management of diseases^5^.

Functional foods are foods or food components that are useful for health beyond basic nutrition. Bioactive compounds present in these functional foods prevent several diseases and thus improve the quality of life^1,6^. Functional foods contain specific minerals, vitamins, essential fatty acids, dietary fibers, antioxidants, probiotics, prebiotics, synbiotics and other bioactive compounds which can provide a specific function to any type of food that contained them^7–9^. Regular consumption of such foods facilitates the effective management of diseases such as cardiovascular disease (CVD), tumor, diabetes and blood pressure, as well as improving memory, mental health, the health of the fetus, losing weight, and reducing the risk of chronic and degenerative diseases, and osteoporosis^6,8,10–13^.

Functional drinks are included in the category of functional foods, which are important sources of vitamins, polyphenols, minerals and dietary antioxidants^14^. Beverages are the most widely used category of functional foods due to the ease of dissolution of functional components and ease of consumption^5,15^. Functional beverage products include dairy beverages (enriched beverages), prebiotic-based beverages, probiotic-containing beverages, non-dairy plant-based beverages made from soy, rice, barley, almonds, or coconut, Grain-based foods, micronutrient-enriched drinks, fortified milk (enriched with calcium, omega-3 and vitamins), fermented drinks such as kefir, fruit juices (enriched with vitamins and omega-3), fortified waters (enriched drinks) with vitamins and minerals, sports and energy drinks, herbal drinks^5,16–18^. Probiotics can be obtained from various sources of cow's milk, i.e. dairy products, as well as from various non-dairy sources such as fruits, vegetables, grains and legumes^19^.

**Figure 1:**
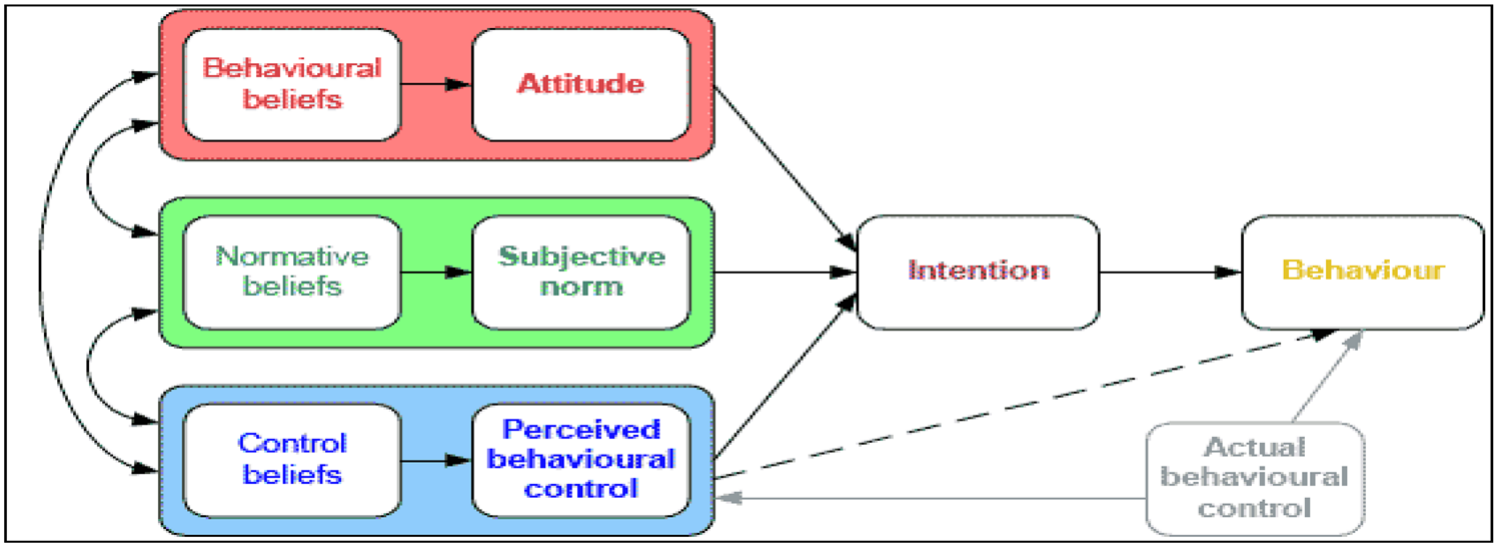
Theory of planned behavior^52^.

Studies show that the behavior of consumers of functional foods is not only dependent on knowledge, but also influenced by demographic factors^2,16,20^. Consumers' perception of the effectiveness of functional foods affects their attitude towards functional foods, and therefore increases their intention to buy functional foods^2,7,16,20^. Increasing consumer awareness and descriptive and supportive norms have a positive effect on the perception of the effectiveness of functional foods^13^. The theory of planned behavior (TPB), suggested by Ajzen (1991), is the most widely used theory in the domain of individuals’ intentions and actual behavior, disease prevention, healthy and sustainable diet, and functional food consumption^21^ (Fig. 1). The TPB has been utilized as a framework to predict individuals’ intention to perform food-related behaviors (e.g., foods enriched with omega-3 fatty acids, fruits and vegetables, sugar-sweetened beverage consumption). According to the TPB, an individual’s intention to participate in a certain behavior is determined by attitudes, subjective norm, and perceived behavioral control^22^. Attitude refers to one’s beliefs regarding the consequences of the behavior. Subjective norm is defined as one’s perceptions of social pressure from significant others to comply with the behavior. Perceived behavioral control is the amount of control one believes they have with regards to completing the behavior^23^. In a meta-analysis study, the TPB not only was reported as the most useful theory to examine intention and behavior, but it was also identified as an appropriate model to develop interventions aimed at improving nutrition^22^.

**Figure 2:**
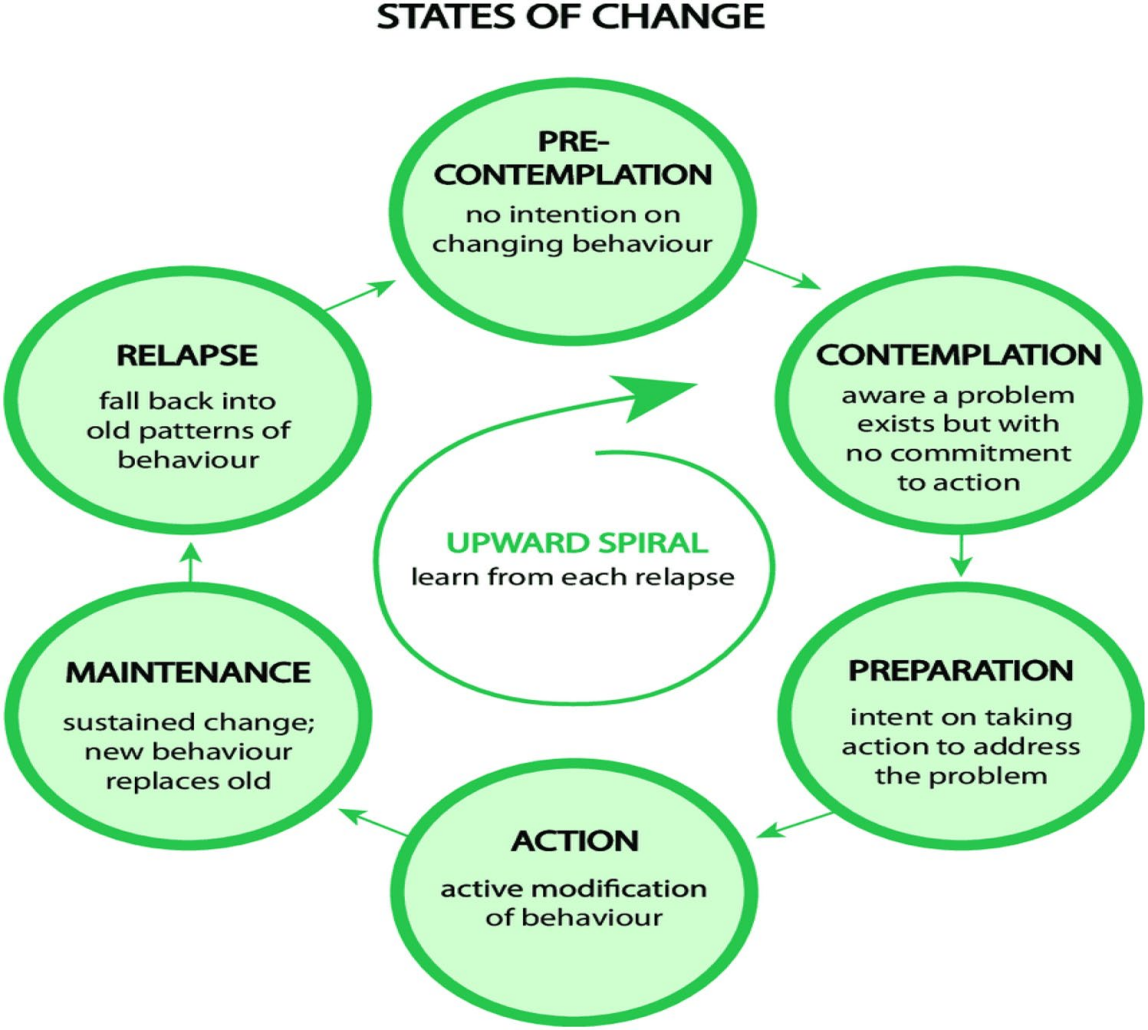
Stages of change model^53^.

Also, Stage of Change (SOC) is one of the most important constructs of Trans-theoretical Model (TTM) that have been shown to be useful in assessing an individual’s readiness to act on health behaviors. Based on this construct, people for adopting a new behavior go through a series of six stages, including: pre-contemplation, contemplation, preparation, action, maintenance, and relapse (Fig. 2). Although TTM includes other key constructs (namely, processes of change, self-efficacy and decisional balance), most of research remains focused on the stages of change construct^24–27^. In this study only the construct of the stages of change for determining the consumption status of functional drinks was used and other constructs of TTM were not investigated.

TPB and the SOC model have been used in various health domains, including eating and drinking behaviors^27–29^. For example, in a study by Armitage et al*.* based on the TPB and the SOC model showed that socio-demographic variables, TPB and attitude change intervention in participants predict the change between most stages of the SOC model^27^. Bakti et al.'s study using the TPB shows that attitude and subjective norm influence the purchase intention of young consumers to buy functional foods. But the perceived behavioral control has no effect on the intention to repurchase and consume functional food^30^.

In recent decades, the importance of women's health and nutrition, especially in the field of diseases and eating disorders, has become significant. Moreover, women’s nutritional requirements change with each life stage^31^. Among the various aspects of promoting women's health and lifestyle, nutritional habits are essential because they apply to all women, can be modified, and affect longevity and quality of life^32^. Most studies reported lower adherence to healthy eating patterns in women^33^. Since women usually allocate a large share of their income to their families, it is considered as one of the channels to improve household living standards, which includes improving nutrition. Women play an important role in determining the family's food plan^34^, due this, the statistical population of this study was selected from women.

The hypotheses of this study were as followed: (1) there is a significant relationship between consumption status of functional drinks and attitude construct of TPB; (2) there is a significant relationship between consumption status of functional drinks and subjective norm construct of TPB; (3) there is a significant relationship between consumption status of functional drinks and perceived behavioral control construct of TPB. According to our information, no study has been conducted to use this model to investigate the behavior of female employees in consuming functional beverages in Iran. The present study was conducted with the aim of investigating the consumption of functional drinks based on the TPB and the SOC model in female employees of Birjand, Iran.

## Results

In this study, 536 female employees in the offices of Birjand were investigated. The mean age of the participants was 40.32 ± 7.6 years. Most of the participants were married (87.9%) and had an income between 50 and 100 million Rials (49.3%), the bachelor's degree (45.7%) consisted the highest proportion of participants education. A small percentage of participants (7.6%) had a special diet and most of the participants (45.5%) purchase from chain stores. About 74.1% of people read food labels and half of the participants (51%) were familiar with functional food (Table 1).

**Table 1:** Frequency distribution of demographic variables in the studied women.

Variable		Std. deviation	Mean
age (years)	Age of participants	7.6	40.32
Number of family member	How many people are there in the family?	1.17	3.90
Number of children	How many children do you have?	1.11	1.74

Variable		Percentage	Number
Marital status	Married	87.9	471
	Single	2.1	65
Level of education	Diploma	3.0	16
	Associate degree	5.2	28
	Bachelor of Art/Science	45.7	245
	Master of Art/Science	39.2	210
	Ph.D	6.9	37
Read food labels	Yes	74.1	397
Familiarization with functional foods (FF)	Familiar with FF	50.9	273
	Knows the definition of FF	60.4	324
Placement in a particular period	Pregnancy	2.2	12
	Breastfeeding	6.3	34
	Menopause	11.9	64
	None	79.5	426
Having a special diet	Yes	7.6	41
family income	Less than 50 million Rial	6.7	36
	50–100 million Rial	49.3	264
	More than 100 million Rial	44.0	236
Place of shopping	Supermarket	35.4	190
	Chain stores	45.5	244
	Special shops	35.8	192
	Local market	41.6	223
	Hypermarket	44.4	238
Participation in the purchase of food	Yes	89.0	477
Responsible in the purchase of food	Yes	37.9	203

**Figure 3:**
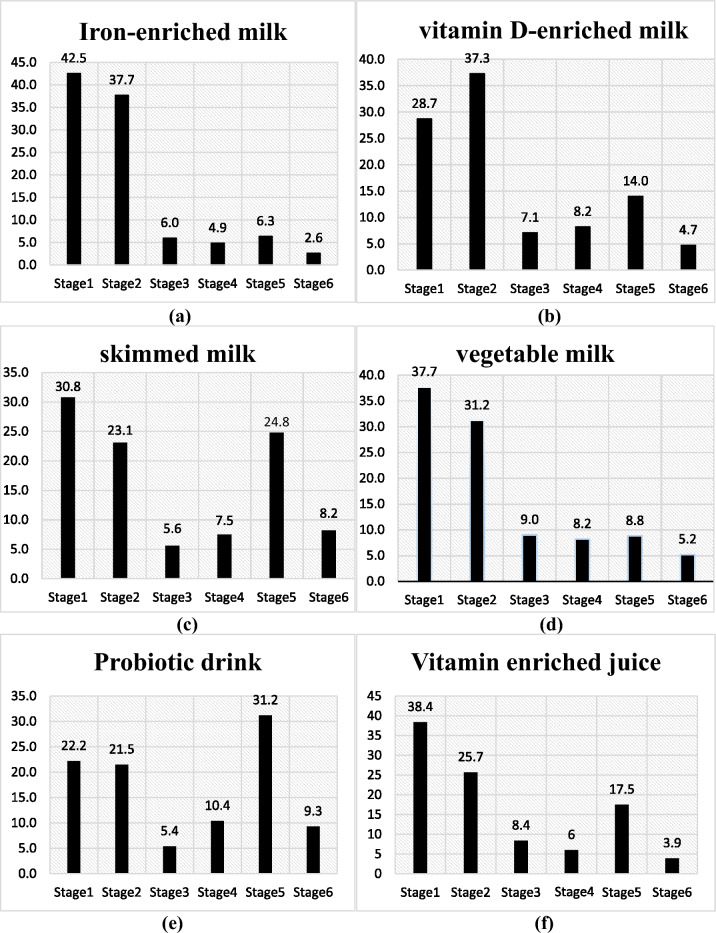
The frequency of stages of change model. X-axis: stages of change model: Stage 1: Pre-contemplation Stage 2: Contemplation, Stage 3: Preparation, Stage 4: Action, Stage 5: Maintenance, Stage 6: Relapse. Y-axis: frequency of stages of change model/Y-axis unit: percentage of choice for consumption.

Based on the construct of SOC, regarding the consumption of iron-enriched milk (Fig. 3a), the highest percentage of people (42.5%) were in the pre-contemplation stage, and the lowest number of participants (2.6%) were in the relapse stage. Regarding the use of milk enriched with vitamin D (Fig. 3b), the largest number of participants (37.3%) was in the contemplation stage and the least number of people (4.7%) were in the relapse stage regarding the consumption of this drink. About the consumption status of vegetable milk (Fig. 3d), most of the participants (37.7%) were in the pre-contemplation stage and the fewest (5.2%) were in the relapse stage (Fig. 3). The mean score of the construct of attitude and subjective norm regarding the consumption of these milks had a significant difference with the stages of change model (*p* < 0.001) (Tables 2 and 3). The highest (30.8%) rate of the participants was about the state of consumption of skim milk (Fig. 3c) in the pre-contemplation phase and the lowest (5.6%) rate was related to the preparation phase (Fig. 3). The mean score of the attitude and subjective norm regarding the consumption of skim milk had a significant relation with the stages of change model (*p* < 0.006 and *p* < 0.001) (Tables 2 and 3).

**Table 2:** Comparison of the mean scores of the constructs of the TPB according to the SOC.

Model constructs	attitude		Perceived behavioral control		subjective norm	
Stages of change	$\overline{x }\pm SD$	m(IQR)	$\overline{x }\pm SD$	m(IQR)	$\overline{x }\pm SD$	m(IQR)
Iron-enriched milk						
Stages I	16.84 ± 3.34^b,c,e,d^	17.00(4.00)	16.84 ± 2.87	17.00(4.00)	20.49 ± 4.61^b,c,e,d^	21.00(6.00)
Stages II	18.12 ± 2.59^a^	18.00(3.00)	16.68 ± 2.87	17.00(4.00)	22.33 ± 3.83^a,e,d^	22.00(4.00)
Stages III	18.56 ± 2.66^a,b^	18.00(3.75)	17.15 ± 2.50	16.50(4.75)	22.25 ± 3.57^a,d^	22.50(4.75)
Stages IV	19.46 ± 2.24^a,b,c^	19.50(3.00)	17.04 ± 2.96	17.00(5.25)	24.50 ± 3.85^a,b,c^	24.00(5.00)
Stages V	18.82 ± 2.93^a,f^	18.50(4.00)	16.82 ± 3.20	17.00(4.25)	23.85 ± 3.67^a,b^	24.00(3.25)
Stages VI	17.14 ± 2.85^e,d^	16.50(5.25)	17.85 ± 2.95	18.50(3.25)	21.57 ± 4.62^d^	22.00(4.50)
*P* value	< 0.001		0.567		< 0.001	
Vitamin D-enriched milk						
Stages I	16.56 ± 3.50^b,c,e,d^	16.00(3.00)	16.56 ± 3.02	17.00(3.00)	20.01 ± 4.61^b,c,e,d^	19.00(5.00)
Stages II	18.00 ± 2.82^a,f^	18.00(4.00)	16.97 ± 2.95	17.00(4.00)	21.99 ± 4.02^a,e,d,f^	22.00(4.00)
Stages III	18.18 ± 2.54^a,f^	18.00(3.00)	17.26 ± 2.23	18.00(3.00)	23.05 ± 3.85^a,f^	23.00(4.00)
Stages IV	18.82 ± 2.46^a,f^	19.00(3.00)	16.90 ± 2.54	17.00(4.00)	23.52 ± 3.38^a,b,f^	24.00(3.00)
Stages V	18.69 ± 2.47^a,f^	18.00(3.00)	16.86 ± 2.98	17.00(4.00)	23.41 ± 3.96^a,b,f^	23.00(6.00)
Stages VI	16.40 ± 2.69^b,c,e,d^	16.00(3.50)	16.52 ± 2.48	16.00(2.50)	19.88 ± 4.03^b,c,e,d^	21.00(4.50)
*P* value	< 0.001		0.498		< 0.001	

a: Significant difference by stage I , b: significant difference by stage II, c: significant difference by stage III, d: significant difference by stage IV, e: significant difference by stage V, f: significant difference by stage VI.

Stage I: Pre-contemplation Stage II: Contemplation, Stage III: Preparation, Stage IV: Action, Stage V: Maintenance, Stage VI : Relapse.

m(IQR): Median)Interquartile Range(

$\overline{x }\pm SD$:Mean ± Std. Deviation.

TPB: Theory of planned behavior.

SOC: stages of change model.

Statistical test: Kruskal–Wallis Test and Mann–Whitney Test.

**Table 3:** Comparison of the mean scores of the constructs of the TPB according to the SOC.

Model constructs	Attitude		Perceived behavioral control		Subjective norm	
Stages of change	$\overline{x }\pm SD$	m(IQR)	$\overline{x }\pm SD$	m(IQR)	$\overline{x }\pm SD$	m(IQR)
Skimmed milk						
Stages I	17.17 ± 3.47^e^	17.00(5.00)	17.74 ± 3.11	17.00(4.00)	20.71 ± 4.85^c,e,d,f^	21.00(6.00)
Stages II	17.64 ± 3.02	18.00(4.75)	16.81 ± 3.06	17.00(4.00)	21.45 ± 4.03^c,e,d^	22.00(5.75)
Stages III	18.80 ± 2.70f.	19.00(4.00)	17.46 ± 3.32	18.00(4.25)	23.40 ± 4.17^a,b,f^	23.50(5.50)
Stages IV	18.47 ± 2.07f.	18.00(3.00)	17.00 ± 2.34	17.00(2.75)	23.20 ± 3.59^a,b,f^	24.00(4.00)
Stages V	18.08 ± 2.37^a^	18.00(3.00)	16.82 ± 2.57	17.00(3.50)	22.62 ± 3.65^a,b,f^	23.00(4.00)
Stages VI	17.09 ± 3.72^c,d^	17.00(5.00)	16.70 ± 2.45	17.00(3.00)	21.09 ± 4.69^a,c,d^	21.00(6.00)
*P* value	< 0.006		0.712		< 0.001	
Vegetable milk						
Stages I	16.92 ± 3.37^c,b,e^	17.00(4.00)	16.68 ± 2.95	17.00(4.00)	20.65 ± 4.75 ^b,c,e,d^	20.00(6.00)
Stages II	17.94 ± 2.92^a^	18.00(4.00)	17.01 ± 2.88	17.00(4.00)	22.18 ± 3.92^a^	22.00(4.00)
Stages III	18.77 ± 2.32^a^	18.00(3.00)	17.37 ± 2.76	17.00(4.00)	22.39 ± 4.15^a^	23.00(4.00)
Stages IV	17.95 ± 2.20	18.00(4.00)	16.72 ± 2.40	17.00(2.00)	22.75 ± 3.2^a^	23.00(3.00)
Stages V	18.53 ± 2.76^a^	18.00(3.00)	16.70 ± 3.07	17.00(3.00)	23.04 ± 3.94^a^	23.00(6.00)
Stages VI	18.00 ± 2.81	18.00(4.00)	16.36 ± 2.80	16.00(3.00)	21.71 ± 4.56	21.50(7.50)
*P* value	< 0.001		0.271		< 0.001	

a: Significant difference by stage I , b: significant difference by stage II, c: significant difference by stage III, d: significant difference by stage IV, e: significant difference by stage V, f: significant difference by stage VI.

Stage I: Pre-contemplation Stage II: Contemplation, Stage III: Preparation, Stage IV: Action, Stage V: Maintenance, Stage VI : Relapse.

m(IQR): Median)Interquartile Range(

$\overline{x }\pm SD$:Mean ± Std. Deviation.

TPB: Theory of planned behavior.

SOC: stages of change model.

Statistical test: Kruskal–Wallis Test and Mann–Whitney Test.

Most of the participants (31.2%) were in the maintenance phase and the least number of ones (5.4%) were in the preparation phase regarding the consumption of probiotic drinks (dough, kefir, etc.) (Fig. 3e). The mean score of the attitude and subjective norm regarding the consumption of probiotic drink with the stages of change model had a significant contribution (*p* < 0.002 and *p* < 0.001) (Table 4). Most of the participants (38.4%) were in the pre-contemplation stage and a small percentage (3.9%) were in the relapse stage regarding the consumption of Vitamin enriched juice (Fig. 3f). The mean score of the attitude and subjective norm regarding the consumption vitamin enriched juice had a significant difference with the stages of change model (*p* < 0.001) (Table 4).

**Table 4:** Comparison of the mean scores of the constructs of the TPB according to the SOC.

Model constructs	Attitude		Perceived behavioral control		Subjective norm	
Stages of change	$\overline{x }\pm SD$	m(IQR)	$\overline{x }\pm SD$	m(IQR)	$\overline{x }\pm SD$	m(IQR)
Vitamin enriched juice						
Stages I	16.82 ± 3.26^c,d,e^	17.00(4.00)	16.55 ± 2.58	17.00(3.00)	20.21 ± 4.55^b,c,d,e^	20.00(5.00)
Stages II	17.97 ± 2.71	18.00(4.00)	17.30 ± 2.92	17.00(5.00)	22.21 ± 3.96^a,d^	22.50(4.25)
Stages III	18.75 ± 2.55^a,f^	19.00(3.00)	17.24 ± 3.12	17.00(4.50)	23.08 ± 3.73^a^	24.00(4.50)
Stages IV	18.50 ± 3.08^a^	19.00(4.75)	16.87 ± 2.73	17.00(4.00)	24.12 ± 4.08^a,b,e,f^	22.50(4.75)
Stages V	18.45 ± 2.80^a^	18.00(3.00)	16.60 ± 3.30	17.00(4.00)	22.34 ± 4.42^a,d,e,f^	24.00(5.50)
Stages VI	17.38 ± 2.85^c^	17.00(4.00)	16.57 ± 2.69	17.00(4.00)	22.89 ± 3.61^d^	23.00(4.25)
*P* value	< 0.001		0.351		< 0.001	
Dough and probiotic drinks						
Stages I	17.12 ± 3.50^c,d,e^	17.00(4.00)	16.98 ± 2.86	17.00(4.00)	20.44 ± 4.68^b,c,d,e^	21.00(6.00)
Stages II	17.54 ± 3.08	18.00(4.00)	17.00 ± 3.04	17.00(4.00)	21.82 ± 4.13^a^	22.00(5.00)
Stages III	18.69 ± 2.07^a,f^	18.00(4.00)	17.20 ± 2.88	18.00(3.00)	22.62 ± 3.68^a,f^	23.00(6.00)
Stages IV	18.51 ± 2.37^a,f^	19.00(3.00)	16.66 ± 2.41	17.00(3.00)	22.76 ± 4.08^a,f^	22.50(4.75)
Stages V	17.96 ± 2.84^a,f^	18.00(4.00)	16.73 ± 2.78	17.00(3.00)	22.34 ± 4.18^a,f^	23.00(4.00)
Stages VI	16.92 ± 3.23^c,d,e^	16.50(4.00)	16.42 ± 3.32	17.00(4.00)	20.78 ± 4.33^c,d,e^	22.00(6.25)
*P* value	< 0.002		0.844		< 0.001	

a: Significant difference by stage I , b: significant difference by stage II, c: significant difference by stage III, d: significant difference by stage IV, e: significant difference by stage V, f: significant difference by stage VI.

Stage I: Pre-contemplation Stage II: Contemplation, Stage III: Preparation, Stage IV: Action, Stage V: Maintenance, Stage VI : Relapse.

m(IQR): Median)Interquartile Range(

$\overline{x }\pm SD$:Mean ± Std. Deviation.

TPB: Theory of planned behavior.

SOC: stages of change model.

Statistical test: Kruskal–Wallis Test and Mann–Whitney Test.

In general, in most of the studied functional drinks with the progress in the construct of the SOC the average of the constructs of attitude and subjective norms have been increased.

Based on the findings of the current study, the mean score of attitude with shopping from hypermarket and chain stores had a significant difference (*p* = 0.01, *p* = 0.05). There was also a significant difference in the mean score of the subjective norm construct in shopping from hypermarkets (*p* < 0.001). Participants who bought more from chain stores had a higher attitude and subjective norm than participants who did not buy from this place. There was a significant difference in the mean score of the subjective norm construct in reading food labels (*p* < 0.002). The mean score of participation in shopping (*p* < 0.002) also showed a significant difference with the construct of subjective norm (Table 5). Participants who tended to read food labels and participate in shopping had a higher subjective norm.

**Table 5:** Comparison of the mean scores of the TPB constructs according to the studied variables.

variable		Attitude		Perceived behavioral control		Subjective norm	
		$\overline{x }\pm SD$	m(IQR)	$\overline{x }\pm SD$	m(IQR)	$\overline{x }\pm SD$	m(IQR)
Buy from the hypermarket	Yes	17.23 ± 3.04	17.00(4.00)	16.87 ± 2.74	17.00(3.25)	20.85 ± 4.31	21.00(6.00)
	No	17.93 ± 3.03	18.00(4.00)	16.81 ± 2.95	17.00(4.00)	22.20 ± 4.27	22.00(6.00)
	*p* value	0.01		0.60		0.001	
Buy from a chain store	Yes	17.91 ± 3.06	18.00(4.00)	17.09 ± 2.83	17.00(3.00)	21.95 ± 4.36	22.00(5.00)
	No	17.50 ± 3.02	17.00(4.75)	16.62 ± 2.90	17.00(3.00)	21.53 ± 4.30	22.00(6.00)
	*p* value	0.05		0.058		0.27	
Read the label	Yes	17.84 ± 2.86	18.00(4.00)	16.97 ± 2.79	17.00(4.00)	22.00 ± 4.24	23.00(5.00)
	No	17.25 ± 3.48	17.00(5.00)	16.43 ± 3.08	17.00(3.00)	20.92 ± 4.49	21.00(6.00)
	*p* value	0.06		0.15		0.002	
Participation in the purchase	Yes	21.89 ± 4.33	22.00(5.00)	16.83 ± 2.91	17.00(4.00)	17.69 ± 3.08	18.00(4.00)
	No	20.35 ± 4.11	20.00(4.00)	16.88 ± 2.58	17.00(2.00)	17.66 ± 2.80	17.00(5.00)
	*p* value	0.68		0.93		0.002	

Statistical test: Kruskal–Wallis Test and Mann–Whitney Test.

## Discussion

Functional foods are foods that by adding certain components to them reduce the risk of disease or improve the health of the body. This study was aimed to determine the consumption status of functional drinks based on the TPB and the SOC Model. TPB and the stages of change model have been used in various health domains, including eating and drinking behaviors^26–28^. Therefore, the status of consumption of functional drinks was investigated based on the theory of planned behavior and the stages of change model in female employees.

### The status of consumption of functional drinks based on the SOC model

According to the results of the current study, in the case of drinks such as iron-enriched milk, vegetable milk and skim milk, most people had not consumed this product and had no desire (intention) to use this product that is in agreement with studies of Rouhani-Tonekaboni et al*.*^35^ and Etehadnezhad et al*.*^36^. Regarding milk enriched with vitamin D, most of the participants had not^6^ used the product, but they had the intention to consume the product (contemplation stage). In the case of vitamin D-enriched juices, most of the participants were in the pre-contemplation stage and they had no intention for consuming this drink. Most of the participants had regular consumption of probiotic drinks (maintenance stage), which was consistent with the study of Kim et al*.*^37^ in relation to a probiotic and functional drink.

The reason for the constant consumption of probiotic drinks may be due to extensive advertising, which has been done by the media or nutritionists. Consumption of plant-based milk and iron-fortified milk was very low among the participants, which may be because of these types of milk that are not well known, less available to the public, or less advertised. It can be also due to people's belief in this matter, the belief that iron enrichment of milk is ineffective due to the interference of iron absorption by calcium. In the study of Etehadnezhad et al*.*^36^, another reason for reducing the consumption of dairy was mentioned due to lactose intolerance in people. In the study of Nolan-Clark et al*.*^38^, a high level of mistrust regarding the effectiveness of functional foods for health was mentioned as a barrier to consumption.

### The status of consumption of functional drinks based on the TPB

Regarding the consumption of functional drinks, all drinks in the attitude construct showed a significant relationship with the SOC model, which is consistent with the studies of O’Connor et al*.*^39^, Salmani et al*.*^2^, Moodi et al*.*^3^, Schnettler et al*.*^40^, Carrillo et al*.*^41^, Urala and Lähteenmäki^42^, Chen^7^, Akhter and Rahut^20^ and Kraus et al*.*^6^. All of these studies are on the same side and conclude that along with the increase of attitude towards the consumption of functional foods, the probability of consumption was increased, and they call the attitude as one of the strong predictors of the consumption of functional foods. It is also in line with the study of Wang and Chu^13^, which showed that the intention of Danish and Norwegian consumers to buy functional foods is influenced by their attitudes towards these foods.

Attitudes are characterized by a person's beliefs about the specific consequences of performing a behavior, such as consuming functional foods, which is measured by evaluating its consequences^43^. Unfortunately, people do not believe in consuming functional foods for various reasons, including distrust of food manufacturers, exposure to conflicting information, fear of unpredictable and dangerous side effects, and distrust of the health claims of these products. Considering that in the case of most of the functional drinks, women employees were in the pre-action stage (pre-contemplation, contemplation, preparation) and considering the effect of attitude on the consumption of functional foods, the dissemination of more information from a reliable source such as health professionals through different communication channels are necessary to increase people's willingness to consume functional foods. In fact, advertising and communication campaigns can influence consumer awareness of functional foods and their health benefits^3^.

Consumption status of drinks also showed a significant relationship with the SOC model in the construct of subjective norms, which are in line with the studies of Salmani et al*.*^2^ and Lalor et al*.*^44^. The results of Wang and Chu's^13^ showed that descriptive and prescriptive norms had an indirect effect on consumers' willingness to buy functional foods through their effect on attitude. In Nystrand and Olsen's study^45^, it is stated that people's attitudes directly affect subjective norms. Subjective norm is determined by one's beliefs about whether important people in one's life approve or disapprove of performing a certain behavior^43^. This normative influence is created by injunctive norms (beliefs about the approval or disapproval of behavior by others) and descriptive norms (beliefs about whether others in one's social networks do this behavior)^23^. Therefore, subjective norms affect the consumer's intention to buy functional foods^13^. The study of Shen et al*.*^46^ also showed that if the influential and important people of consumers have positive opinions and attitudes towards a type of food, consumers will most likely have a high intention to buy that type of food. In order to influence people's perception, producers of functional foods can use important public figures (celebrities) and encourage people to consume this type of food. The chosen celebrity should be someone who represents a healthy lifestyle. Bakti et al.'s. study^30^ also stated that family members can influence each other in the consumption of functional foods.

Results of present study showed that functional drinks in the behavioral control construct did not show a significant relationship with the SOC model, which is consistent with the studies of Bakti et al*.*^30^, Salmani et al*.*^2^, Patch et al*.*^47^ and O'Connor, and White^39^, however, the results of the studies by Moodi et al*.*^3^ and Xin et al*.*^48^ showed that perceived behavioral control is significantly related to the consumption of functional foods. Perceived behavioral control is a person's perception of the ease or difficulty of a given behavior. This perception is determined by people's beliefs regarding the existence of factors that may make it easy or difficult to perform a certain behavior^43^. The high price of a profitable product compared to a similar non-profitable product and people's low confidence in the effectiveness of these products can be an obstacle to buy this product. A large percentage of the studied women were also married, which may influence the decision and desire of their husbands to buy food.

### Demographic information based on the TPB

Based on the results of the current study, place of shopping, reading the food label, participation in the food shopping, and the amount of family income showed a significant difference with the constructs of the TPB. Most people's purchases were from chain stores and hypermarkets. Buying from hypermarket had a significant difference in the constructs of attitude and subjective norm. Buying from chain stores had also a significant difference in attitude, which was in line with the studies of Hong et al*.*^49^ and Salmani et al*.*^2^.

This study showed that people who the highest consumption of functional foods had bought from chainstores, which may be due to the abundance of this type of food in these shopping centers. Also, the abundance of other consumer products that lead to saving time for buyers to go to other shopping places may be effective. Sometimes, in this type of stores, there are representatives from food companies who explain the type of food, which in some way affect the awareness and attitude of the buyers. Hypermarkets also create the impression that they are suitable for providing various products^49^. The high variety of products that may be due to the large space of the chainstore is also effective in attracting the attention of people to buy from this place. It is also possible that the satisfaction of buying different food items and other non-food products at the same time and place leads to recommendations to friends and neighbors, which has an impact on subjective norms.

In this study, label reading showed a significant relationship in the construct of subjective norms, which was in line with the study of Tian et al*.*^50^. In Tian et al.'s. Study, attitudes, descriptive norms, and nutrition literacy were significant predictors of food label usage intentions. Coulson study^25^ showed that a higher percentage of people who always read the food label read were in the maintenance stage. The results of our study showed that friends and important people in a person's life can play a significant role in reading food labels and drawing attention to functional foods and drinks and increasing the consumption of functional foods. Lack of shopping time and the ability to understand food labels (nutrition literacy) are also important in label reading. In Nolan-Clark et al.'s. study on normative beliefs, it was shown that these beliefs are more stable and less inclined to change behavior than control and behavioral beliefs through nutrition education^38^, therefore, improving nutrition literacy increases label reading and the effect of subjective norms causes permanence behavior. In other demographic variables such as education, income, number of children, etc., there was no significant difference with the model constructs, which is in line with Babicz-Zielinska and Jezewska-Zychowicz's study^51^ and Moodi et al.'s. Study, it was stated in this study, Ozen, Pons, and Tour (2012) systematically reviewed twenty-three studies around the world on the consumption of functional products belonging to different food categories. They concluded that it was not possible to distinguish how education level and socio-economic characteristics clearly affect the consumption of functional foods^3^.

However, in the results of other studies, these demographic variables were significantly different, such as the study of Kim et al*.*^37^ in which the effect of gender, education, geographic region and culture were significant.

### Limitations

This study is not without limitations. First, our study was cross-sectional and the long-term effect was not investigated, which with the increase in women's awareness of the consumption of functional foods the consumption’s status may be changed.

In the study, only the construct of the SOC of the TTM model was used, and other constructs of the TTM were not investigated. Other constructs can be helpful in investigating the reasons for the reduction or lack of consumption of functional foods and can also be effective in helping to plan educational interventions. Also, the questionnaires were completed by the people themselves, and there was a possibility of errors in completing the form.

## Conclusion

The first and second hypotheses of the current study were confirmed but, the third one was not confirmed.

According to the findings, the attitude and subjective norms of people are the main determinants of the consumption of functional drinks. The results of present study showed that due to the fact that half of the people are familiar with functional foods, most of them are in the pre-contemplation stages and have very low consumption of these foods, which should be further investigated and other main factors influencing the choice behavior.

The participants who are in the pre-contemplation, contemplation and preparation stage have a higher attitude towards consuming drinks, and in other words, by passing from the pre-contemplation stage to contemplation and preparation, the attitude and subjective norms of people were increased.

Due to the high importance of these drinks, it is possible to identify people in different stages of behavior change and by educating people (people who are in the stages before action and relapse) about the benefits of consuming functional foods and increasing awareness caused a change in the attitude of family members and influenced the subjective norms of people.

Also, the findings of this study have helped to design health interventions by health education specialists, which can be an effective step towards improving the health of individuals and society. The findings of this study will be valuable for marketers in the functional food industry to create marketing communication strategies and facilitate the development of this industry. It is better to conduct more studies in order to confirm the influence of the TPB constructs on the SOC Model.

## Methods

### Study design and participants

The current research was a cross-sectional descriptive-analytical study that was conducted in the spring of 2022 on the female employees in the offices of Birjand, Iran. The number of samples was calculated using the following formula according to the study by Salmani et al.^2^.The sample size was calculated as 441 people with considering 20% downfall, at least 536 people were considered.

$$n = \frac{{\left( {z_{{1 - \frac{\alpha }{2}}} + z_{1 - \beta } } \right)^{2} \left( {\sigma_{1}^{2} } \right)}}{{d^{2} }} = \frac{{\left( {1.96 + 0.84} \right)^{2} \left( {0.75^{2} } \right)}}{{0.088^{2} }} \approx 441$$

In this study, we used multi-stage sampling. The offices had different number of employed women, some had fewer and some had more. Therefore, the offices were classified based on the number of employed women, initially in categories of 10–20, 20–30,… , 100–200. Then, the sample size was determined proportional to the size of each category based on the total population of employed women. Next, the offices were randomly selected from each category, and employed women were selected from the convenience sampling. The sampling continued until the allotted sample size for each classify was reached. Inclusion criteria were filled informed consent and desire to participate in the study, being employed in one of the offices of Birjand, and the exclusion criterion was incomplete completion of the questionnaire.

### Study instrument

The research tool was a standard questionnaire^2^. The questionnaire consisted of three parts of demographic information, the construct of TPB, and the construct of the stages of change. The demographic information section included 19 questions related to characteristics such as age, marital status, level of education, number of family members and children, family income, shopping place, reading food labels, etc. The second part of the questionnaire was consisted of 16 questions (attitude 5 question, behavioral control 5 question, and subjective norms 6 question) with 5 point Likert scale. The validity and reliability of the questions in this research as well as the research^2^ from which the questionnaire was taken have been confirmed. The third part of the questionnaire was in the form of one question related to SOC. In order to measure the construct of intention and behavior, the construct of the stages of change model has been used.

The questions of the change stages model examined 6 stages: pre-contemplation, contemplation, preparation, action and maintenance and relapse, and according to the situation explained about the stages of change, people chose only one option (one stage) for each drink. Choosing the pre-contemplation stage and the relapse stage means that the person did not intend to consume functional drinks, and the contemplation and preparation stages mean that having an intention to consume, and selecting the action and maintenance stages mean the consumption behavior in the participants. The validity of stage of change construct was confirmed with expert panel (CVI = 1, CVR = 1) and the reliability with test–retest method was approved by kappa > 0.80.

It should be mentioned that the drinks considered in this study include: iron-enriched milk, vitamin D-enriched milk, fat-free milk, plant-based milks such as soy milk, almond milk, dough and probiotic drinks, kefir and vitamin-enriched juices.

### Statistical analysis

After collecting the data, it was recorded in SPSS (version 19) and described using descriptive statistics for quantitative variables (mean and standard deviation, median and interquartile range) and for qualitative variables (number and percentage). To check the assumption of normality, Kolmogrov-smirnov test was used. Independent t-test (non-parametric equivalent was Mann–Whitney) and one way analysis of variance (ANOVA) (Kruskal–Wallis was non-parametric equivalent) with Tukey's post hoc test were used to compare two and more quantitative groups at significance level (*P* < 0.05).

### Ethical approval and consent to participate

This study was confirmed by the ethical committee and institutional review board of Birjand University of Medical Sciences (IR.BUMS.REC.1401.086). Filling of the questionnaire was voluntary and anonymous by the respondents. Written Informed consent was obtained from all participants. All methods were performed in accordance with the relevant guidelines according to the Declaration of Helsinki.

## Data Availability

The datasets generated and/or analyzed during the current study are not publicly available but are available from the corresponding author on reasonable request.
